# Early diagnosis of colorectal cancer using Cerenkov luminescence endoscopy: a pilot trial involving humans for the first time

**DOI:** 10.7150/thno.122007

**Published:** 2026-01-08

**Authors:** Ze Yang, Zhuojun Wu, Tiantian Pang, Dan Liu, Xinyu Wang, Jingmin Yu, Shicheng Xu, Xiaoyu Kang, Dacheng Liao, Zuhong Tian, Yunhu Bai, Xiaojuan Xi, Tianyu Yan, Xiaojian Lu, Yu Qi, Mingru Zhang, Lina Zhao, Fei Kang, Shuhui Liang, Jing Wang, Xueli Chen, Kaichun Wu

**Affiliations:** 1 State Key Laboratory of Holistic Integrative Management of Gastrointestinal Cancers and National Clinical Research Center for Digestive Diseases, Xijing Hospital of Digestive Diseases, Fourth Military Medical University, Xi'an 710032, China.; 2 Department of Gastroenterology, 967th Hospital of the PLA Joint Logistic Support Force, Dalian, Liaoning 116021, China.; 3 Northwestern Polytechnical University, Xi'an 710072, China.; 4 Engineering Research Center of Molecular and Neuro Imaging of Ministry of Education & School of Life Science and Technology, Xidian University, Xi'an 710071, China.; 5 Department of Medical Engineering, 967th Hospital of the PLA Joint Logistic Support Force, Dalian, Liaoning 116021, China.; 6 Department of General Surgery, 988 Hospital of Joint Logistic Support Force, Zheng Zhou 450000, China.; 7 Nanjing Chunhui Science and Technology Industrial Co. Ltd., Nanjing 210012, China.; 8 Department of Nuclear Medicine, Xijing Hospital, Fourth Military Medical University, Xi'an 710032, Shaanxi, China.; 9 Department of Radiation Oncology, Xijing Hospital, Fourth Military Medical University, Xi'an 710032, Shaanxi, China.

**Keywords:** Cerenkov luminescence imaging, Cerenkov luminescence endoscopy, molecular imaging, endoscopic diagnosis, early colorectal cancer

## Abstract

**Rationale:** Current gastrointestinal endoscopy mainly depends on morphological changes for lesion diagnosis, thus often failing to detect early colorectal cancers (CRCs) with subtle morphological alterations. Optical molecular imaging via endoscopy may provide a unique means to identify early CRCs that precede the morphological changes observed via conventional endoscopy. In addition, optical imaging methods are utilized for intraoperative navigation when imaging tumors. However, the primary challenge in applying optical molecular imaging clinically is the restricted kinds of clinically endorsed targeted probes. Cerenkov luminescence (CL) can be observed with almost all clinically validated radiotracers. Therefore, Cerenkov luminescence imaging (CLI) does not require the development of new probes and can directly utilize clinically validated radiotracers. This study aimed to use Cerenkov luminescence endoscopy (CLE) for diagnosing early CRC effectively.

**Methods:** In a prospective observational study, we use a self-produced CLE to diagnose colorectal lesions (mainly CRC). The CL images of the lesions were recorded and analyzed in comparison with PET/CT scans and histopathology.

**Results:** A total of 20 colorectal lesions from 15 patients were included in the study. The agreement between CLE and PET/CT in diagnosing early CRC (stage Ⅰ CRC and advanced adenoma) was 100%. The level of agreement of CLE images with histopathology was 88.9% acceptable to high for early CRC. Compared with that of colorectal hyperplastic polyps, the signal-to-background ratio of CLE from early CRCs was significantly greater (1.33 ± 0.17 vs 0.99 ± 0.03, *P* < 0.001). In phantoms, tumor-bearing nude mice, and rectal pseudotumor model dogs, CLE detected CL at the corresponding lesion locations.

**Conclusions:** This study demonstrated for the first time that CLE could utilize Cerenkov luminescence molecular imaging to diagnose early CRCs, overcoming the limitations of current endoscopic diagnosis based on morphological changes. (ClinicalTrials.gov, NCT05575765).

## Introduction

The incidence and mortality rates of colorectal cancer (CRC) are the highest among malignant tumors of the digestive system, with approximately 1.9 million new cases and 900,000 deaths per year 1. The prognosis for patients with CRC is closely linked to the stage of their disease, and the survival rate after five years for patients with early-stage CRC can attain 90%, in contrast to the figure for those with advanced CRC, which is below 14% 2.

Gastrointestinal endoscope has long been an important tool for diagnosing early CRCs. By endoscopic screening of early CRC including advanced adenomas, the mortality of CRC decreased in many countries 1, 3. Despite the increasing number of improvements in gastrointestinal endoscopes, early diagnosis of CRC is far from ideal 2-4. Conventional white light endoscopy has a high rate of missed diagnoses for colorectal lesions. The diagnostic performance of computer-aided detection technologies, which have emerged in recent years, does not outperform white light endoscopy for the diagnosis of colorectal lesions in real-world settings, and they also face issues such as a high rate of false-positive identifications 5, 6. Image-enhanced endoscopies, such as Narrow Band Imaging and flexible spectral imaging color enhancement, while aiding in the real-time determination of the pathological nature of colorectal lesions, do not improve the detection rate of colorectal lesions compared to white light endoscopy 7. In summary, current endoscopic diagnoses are based mainly on morphological changes in lesions and often fail to detect early CRCs with inconspicuous morphological changes. The molecular alterations within tumor cells occur well before visible morphological changes can be observed 8, 9, and molecular imaging can improve the early diagnosis of CRC. PET is an important molecular imaging modality that can accurately identify tumors, and recent studies have demonstrated its feasibility for real-time guidance of tumor therapy or biopsy 10, 11. However, PET requires large and complex dedicated imaging equipment, with merely 10% of countries possessing at least one scanner per million people, and the number of PET dedicated specifically to image-guided interventions is even smaller 10. In addition, PET examinations are costly, and the spatial resolution of PET remains limited 12, 13. By contrast, the hardware required for optical molecular imaging is far simpler, more portable, and less costly, and the imaging operation is also more convenient, with higher resolution for optical imaging 10. These advantages make optical molecular imaging one of the most widely adopted molecular imaging techniques 14, 15.

Traditional optical contrast agents, such as sodium fluorescein and indocyanine green, lack tumor-targeting capabilities 16, 17. In recent years, several new types of optical probes with targeted effects on tumors have been reported 18, 19. However, most of these optical targeted probes have not been approved by regulatory authorities for clinical use and thus cannot be officially introduced into clinical practice. Currently, the major obstacle to the clinical application of optical molecular imaging lies in the limited number of formally approved optical targeted probes 20-22.

Cerenkov luminescence imaging (CLI) represents an innovative and distinctive optical technique that has garnered attention in recent years. Cerenkov luminescence (CL) occurs when charged particles move through a medium at a speed greater than the speed of light in that medium. The decay of radioactive isotopes produces CL via the emission of charged particles that achieve super-luminal velocities in a medium. CL can be observed with almost all clinically validated radioisotope probes. Therefore, CLI does not require the development of new probes and can directly utilize clinically validated radioactive isotope probes 23. The key is a facility to detect CL efficiently in the body, which is not commercially available. Our group has successfully developed and verified a system of Cerenkov luminescence endoscopy (CLE) with high detection sensitivity in vitro 24. However, this endoscopic system has not yet been used in vivo to detect lesions of the digestive tract, including CRCs, and its clinical value remains to be verified.

The objectives of this study were as follows: (1) to use a self-developed CLE system to achieve diagnosis via molecular imaging in patients with early and advanced CRC; (2) to evaluate the accuracy of CLE imaging from both histological and PET/CT perspectives; (3) to use the CLE system to differentiate patients with early CRC from those with benign polyps; and (4) to validate the localization of the CL in three preclinical models: phantoms, tumor-bearing nude mice, and dogs.

## Methods

### Construction of the combined CLE system

Previously, we independently developed a single-modal CLE with high detection sensitivity by optimizing the structural parameters of the fiber endoscope and connecting the endoscope to an electron-multiplying charge-coupled device (EMCCD) camera (Andor iXon Ultra 897, Oxford Instruments, UK), which can detect nuclides at the submicrocurie level in less than 300 s 24. In addition, we have integrated the single-modal CLE with a high-resolution electron white light endoscope to construct a new dual-modal CLE, where each individual endoscope is imaged with a separate channel, reducing the influence of each other and allowing simultaneous CL imaging and electron white light imaging. The new CLE can almost acquire the all-spectrum CL.

### Patient characteristics

We recruited patients with colorectal cancers, advanced adenomas or hyperplastic polyps from Xijing Hospital between November 2022 and April 2024. The inclusion criteria for patients were as follows: (1) age > 18 years; (2) histopathologically confirmed CRCs, advanced adenomas, or hyperplastic polyps; (3) no allergy to the relevant imaging agents; (4) ability to understand and sign the informed consent form; and (5) willingness to participate in this study. The exclusion criteria for patients were as follows: (1) previously treated with any therapy (endoscopy, surgery, targeted therapy or radiotherapy, etc.) for colorectal lesions; (2) a history of severe, progressive, or uncontrolled renal, hepatic, gastrointestinal, endocrine, pulmonary, cardiac, or neurological diseases; (3) currently pregnant or breastfeeding; or (4) currently have no personal freedom or independent civil capacity.

### Procedures

All patients fasted on the examination day, and polyethylene glycol electrolyte dispersions were administered to induce diarrhea until pale yellow, watery stools with no solid fecal material passed. An intravenous injection of [¹⁸F]FDG/[⁶⁸Ga]Ga-FAPI-04 (0.1 mCi/kg) was administered approximately 50 minutes before CLE examination. After the patient was placed in the left lateral position, the endoscope was pushed through the anus into the lumen of the bowel after 10 mg of anisodamine was administered intramuscularly. After identifying the colorectal lesion, the initial step involved capturing a white light image. This image was taken using the illumination provided by the endoscopic light source. The purpose of this image was to establish an anatomical reference. It was essential for the field of view to encompass not only the lesion itself but also the adjacent normal tissues. The endoscope was then kept still with the same view for a maximum of 5 minutes of CL imaging while the endoscopic light source was turned off. Immediately after CL imaging, the patient underwent a standard PET/CT scan (uMI 780, United Imaging). The endoscopist and the nurse wore a personal alarm dosimeter (RAD-60, Mirion, Finland) on their chests at the beginning of the CL imaging session. After completing one imaging session with the CLE system, the personal alarm dosimeter was removed, and the readings were recorded.

### Image analysis

After image acquisition, the signal intensity was measured in areas of the lesions (signal) and surrounding normal tissue (background), and the signal-to-background ratio (SBR) was calculated. The localization of all malignant lesions (histologically confirmed CRC and advanced adenomas) in CL images vs white light images was scored for consistency by two independent endoscopists via the Likert scale, and a third experienced endoscopist made a judgment when there was conflict. Two independent nuclear medicine physicians interpreted the PET/CT results. If there was any disagreement between the two physicians, a third experienced nuclear medicine physician made a judgment. The concordance between the CL and PET/CT images for diagnosing malignant lesions was evaluated.

### Related definitions

CRC and surrounding normal tissue were determined based on endoscopic findings and histopathology. The histopathology of tumor was referenced according to the WHO classification of digestive system tumors. Surrounding normal tissue refers to the healthy tissue around the tumor site that has not been invaded by tumor cells and still maintains normal structure 25.

An advanced adenoma is defined as an adenoma with high-grade intraepithelial neoplasia, a villous component, and/or a diameter greater than 10 mm.

The CL signal strength in the region of interest was determined by subtracting the intensity of the background signal in the area without nuclides from the raw signal intensity explicitly recorded in the target area.

### Sample size

Since this study is exploratory research and lacks prior baseline data for reference, the sample size for the clinical research part is mainly assessed from the perspective of practical feasibility, with an estimated inclusion of 15 participants.

### Statistical analysis

The analysis of the data was conducted using GraphPad Prism (version 8.3.0). Continuous data are reported as the mean ± standard deviation. The agreement of CL intensity between the two imaging systems was fit with a Pearson correlation. Group comparisons for quantitative data were conducted using either a two-tailed t-test or a Mann-Whitney test; comparisons between groups of qualitative data were performed via Fisher's exact test; Statistical significance was set at a *P*-value of less than 0.05.

A comprehensive description can be found in the supplementary material.

## Results

Between November 2022 and April 2024, 16 patients were included in the study. One patient had to be excluded due to an imaging equipment malfunction, leaving 20 lesions in 15 patients for analysis. The cohort comprised 20 lesions including stage I-IVB colorectal cancer (AJCC stages) (11 cases), colorectal adenomas (5 cases), and hyperplastic polyps (4 cases). (Details of lesion characteristics are listed in Table S2).

### Construction and characterization of a combined CLE system

**Figure 1:**
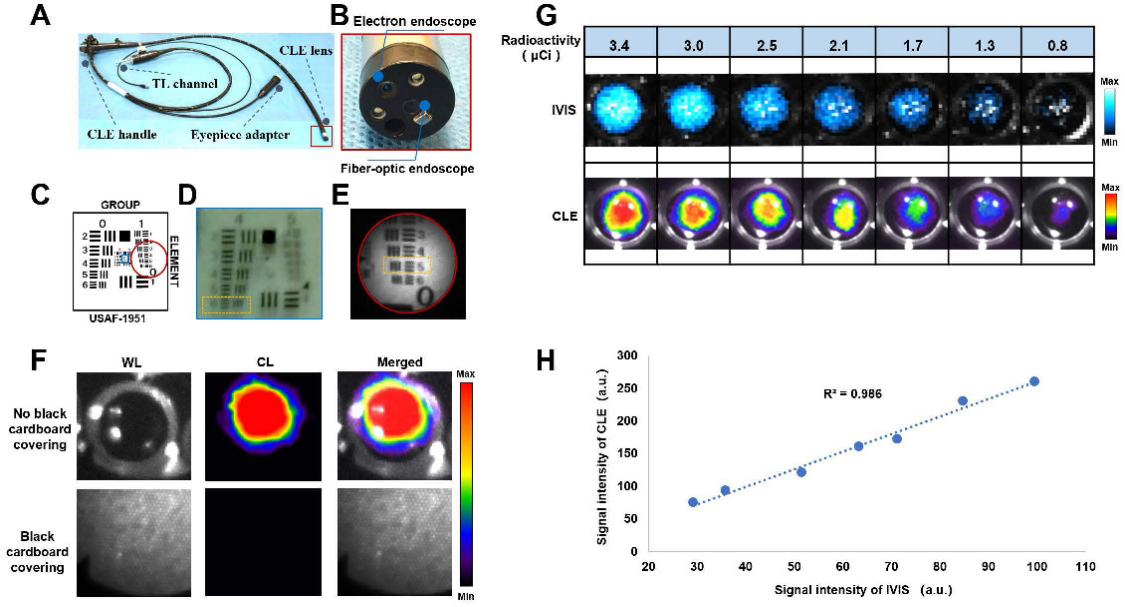
** Characterization of a combined CLE system**. Overview of CLE (A). CLE lens components (B). Schematic diagram of the standardized resolution test target (USAF 1951, USA), where the blue box indicates the field of view of the electronic endoscope, and the red circle indicates the field of view of the fiberoptic endoscope (C). Characterization of the electronic endoscopic resolution of the CLE system (the orange dashed box shows the minimum line spacing that the electron endoscope of the CLE system could recognize) (D). Characterization of the fiber-optic endoscopic resolution of the CLE system (the orange dashed box shows the minimum line spacing that the fiber-optic endoscope of the CLE system could recognize) (E). CL confirmation experiments (F). Images (G) and linear fitting results (H) of the signal intensity of the CLE system and a commercial optical imaging system (IVIS Lumina S5) when multiple sets of ^68^Ga with the same radioactivity were used as the light source under the same imaging time and binning value. TL, transmission light; CLE, Cerenkov luminescence endoscopy; WL, white light; CL, Cerenkov luminescence.

We constructed a combined CLE system by integrating an electronic endoscope and a fiber-optic endoscope (Figures 1A-B). The main characteristics of the CLE system are shown in Table S1. An electronic endoscope can acquire color RGB images under light conditions for morphological observation of lesions. The fiber-optic endoscope allows the acquisition of the CL from the lesion, which is used for molecular imaging of the lesion via an EMCCD camera. Resolution testing was performed on the basis of a standardized resolution test target (Figure 1C). The electron endoscope could resolve up to line pairs of group four, element six (Figure 1D), which was calculated according to equation with a maximum resolution of 0.035 mm 26. The fiber optic endoscope could resolve up to line pairs of group one, element five (Figure 1E), which was calculated according to equation with a maximum resolution of 0.315 mm 26. CL confirmation experiments revealed that the light emitted from the nuclide holes was visible when there was no black cardboard covering. However, the light emitted from the nuclide holes could not be detected when black cardboard covered the holes, thus verifying that the CLE system was detecting CL rather than scintillating luminescence caused by gamma rays, which can easily penetrate black cardboard (Figure 1F). The CLE system was compared with a commercial optical imaging system (IVIS Lumina S5, PerkinElmer, USA) using the same radioactivity of ^68^Ga under the same testing conditions. The results showed that the CLE had a good correlation with IVIS Lumina S5, with R^2^ = 0.986, which indicated that the measurement results of the CLE system were stable (Figures 1G-H).

### CLE for the diagnosis of CRCs (stage I-IV)

**Figure 2:**
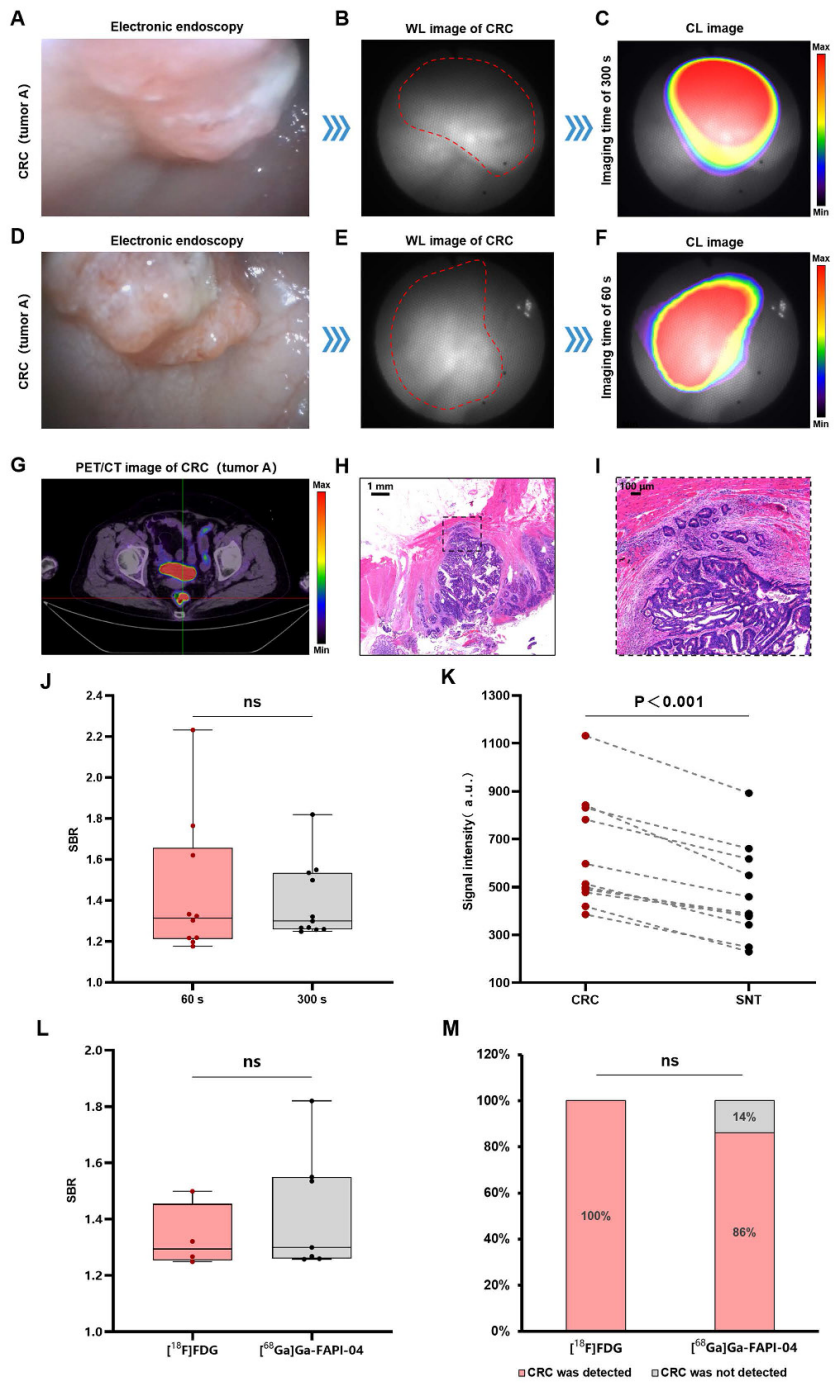
** CLE imaging of CRC**. A representative image of electronic endoscopy for CRC (stage IIA rectal cancer referred to as tumor A) (A). WL photograph of tumor A (red dotted line) (B). CL image ([¹⁸F]FDG) of tumor A (imaging time of 300 s) (C). The imaging process of rectal cancer by CLE ([¹⁸F]FDG) with an imaging time of 60 s (D-F) (another view of tumor A). PET/CT image ([¹⁸F]FDG) of tumor A (G). Pathology images (H-I) of tumor A. Comparison of the SBRs of CL images with image acquisition time of 300 s and 60 s (Mann-Whitney test, J). Comparison of CL signal intensity between CRC and SNT (t-test, n=11, K). Comparison of the SBRs of CL images of [¹⁸F]FDG and [⁶⁸Ga]Ga-FAPI-04 (t-test, L). Comparison of CL images of [¹⁸F]FDG and [⁶⁸Ga]Ga-FAPI-04 to detect CRC (Fisher's exact test, M). CLE, Cerenkov luminescence endoscopy; CRC, colorectal cancer; WL, white light; SNT, surrounding normal tissue; CL, Cerenkov luminescence; ns, not significant.

The CLE system could detect CL in the colorectal cancer region, and this signal corresponded precisely to the lesion's anatomical location as visualized in the white-light image. Figure 2A shows an electronic endoscopic image of a patient with stage IIA (T3N0M0) rectal cancer (named as tumor A). Figures 2B-C show the CL imaging results of the patient with a 300 s image ​​acquisition time​​. PET/CT revealed the same localization result (Figure 2G), confirming the accuracy of CL imaging. To enhance clinical feasibility, we reduced the image acquisition time to 60 s. Figure 2D-F show the imaging process of rectal cancer by CLE with a 60 s image acquisition time, and the area with an abnormally high signal still coincides with the location of rectal cancer (another view of tumor A). The SBRs were comparable between the 300 s and 60 s (1.39 ± 0.18 vs 1.44 ± 0.34, *P* = 0.81) (Figure 2J). The histology of a moderately differentiated adenocarcinoma of the rectum is shown for the patient (Figures 2H-I). Quantitative data analysis of all of the CRC patient samples (n = 11) revealed that the CL signals of the CRC samples were significantly greater (*P* < 0.001) compared to the surrounding normal tissue samples (Figure 2K). CLE identified 91% of the CRCs (10/11), PET/CT had a 100% CRC detection rate (11/11), and the agreement between CLE and PET/CT for the diagnosis of CRC was 91% (10/11). In addition, we compared the imaging quality of two contrast agents, [¹⁸F]FDG and [⁶⁸Ga]Ga-FAPI-04, and there was no significant difference between the SBRs of the images of the two contrast agents (1.33 ± 0.11 vs 1.43 ± 0.22, *P* = 0.45) (Figure 2L). In terms of the CRC detection rate, the [¹⁸F]FDG level was 100% (4/4), and the [⁶⁸Ga]Ga-FAPI-04 level was 86% (6/7), which was not significantly different (*P* > 0.99) (Figure 2M).

### CLE for the diagnosis of early CRCs (stage I CRC and advanced adenoma)

**Figure 3:**
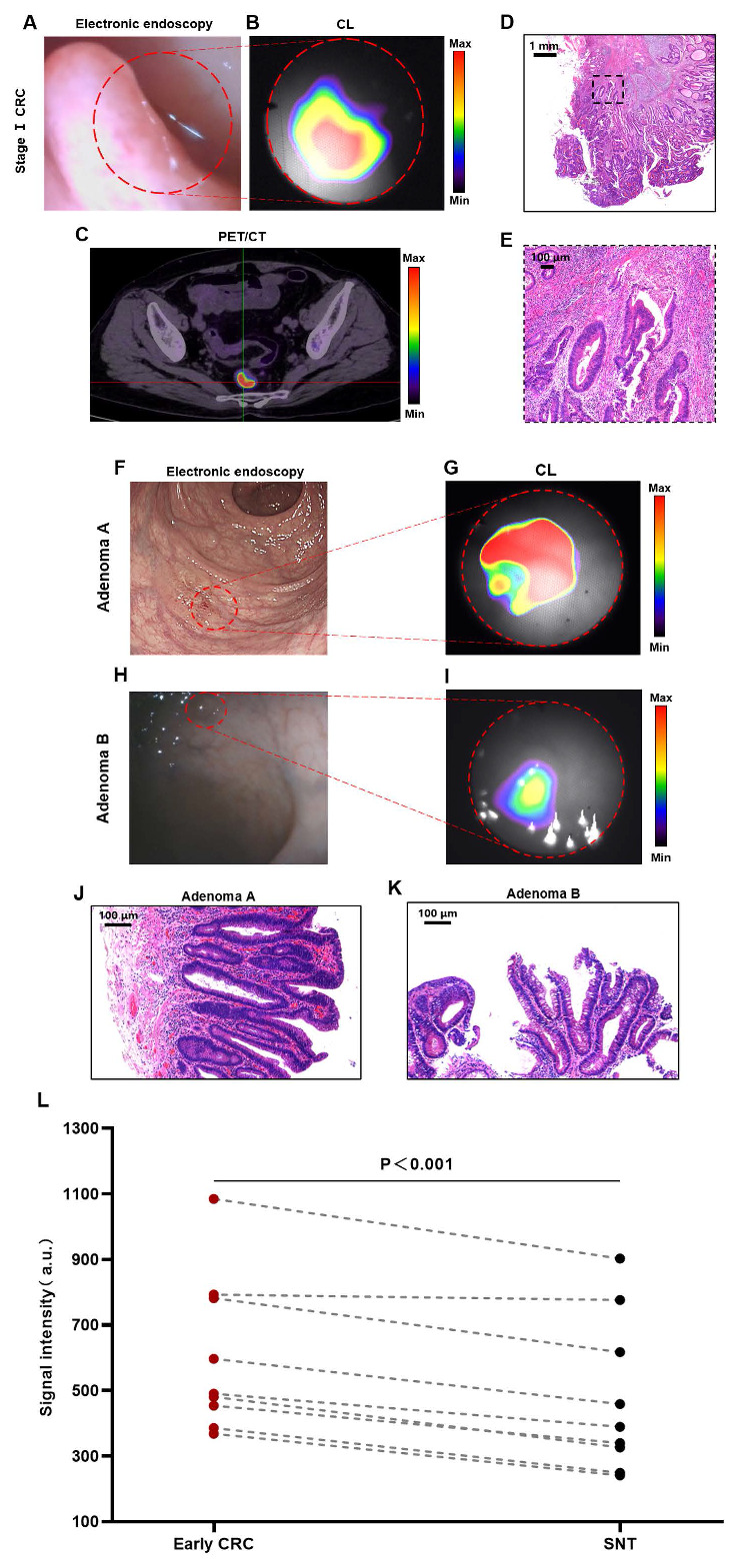
** CLE imaging of stage Ⅰ CRC and advanced adenoma**. Representative images of CLE ([¹⁸F]FDG) in stage I CRC (red dotted line) (A-B). PET/CT photograph ([¹⁸F]FDG) of stage I CRC (C). Pathology images (D-E) of the stage I CRC patient. Representative images of CLE ([⁶⁸Ga]Ga-FAPI-04) in adenomas (red dotted line) (F-G, H-I). Pathological images (J-K) of the adenomas. ​​Comparative analysis of CL intensity contrasting early CRC lesions with the SNT (t-test, n=9, L). Early CRC includes stage I CRC and advanced adenoma; CLE, Cerenkov luminescence endoscopy; CRC, colorectal cancer; CL, Cerenkov luminescence; SNT, surrounding normal tissue.

Early diagnosis plays a crucial role in enhancing the prognosis for CRC patients, so we further analyzed CLE imaging of stage I CRC patients. Figures 3A-B show the imaging results of a patient with stage Ⅰ rectal cancer (T1N0M0). The CLE system could detect CL in the rectal cancer region, and this signal corresponded precisely to the lesion's anatomical location as visualized in the white-light image. PET/CT revealed the same localization result (Figure 3C), confirming the accuracy of CL imaging. The histology of a moderately differentiated adenocarcinoma of the rectum is shown in Figures 3D-E.

Adenomas are the most common precancerous lesions of CRC, and the adenoma detection rate (ADR) by colonoscopy is the most efficient means of screening for CRC 27, 28. In this study, we defined advanced adenoma and stage I CRC together as early CRC. Figures 3F-G show the detection of a case of flat adenoma (adenoma A) with the CLE system. Electron endoscopy revealed flat mucosal elevation with a color similar to that of the surrounding mucosa, which was easy to miss. In contrast, when imaging by the CLE system, the lesion presented a clear high-signal area that was different from the surrounding normal tissue. The rectal tubulovillous adenoma of the patient's histology is shown in Figure 3J. Figures 3H-I show the detection of a small adenoma (adenoma B) by the CLE system. Electron endoscopy revealed a polypoid lesion approximately 0.2-0.3 cm in size with a color similar to that of the surrounding mucosa, which was easy to miss. However, imaging by the CLE system could differentiate lesions with a clear area of high signal from the surrounding normal tissue. A tubulovillous adenoma of the rectum was confirmed by histology (Figure 3K).

Quantitative data analysis of all patients with early CRC revealed that the CL signals from early CRCs were significantly greater than those from the surrounding normal tissue (*P* < 0.001) (Figure 3L). Both CLE and PET/CT identified 88.9% of early CRCs (8/9). The agreement between CLE and PET/CT in diagnosing early CRC was 100% (including 8 true positive cases and 1 false negative case, 9/9).

### CLE for differentiating benign and malignant colorectal lesions

**Figure 4:**
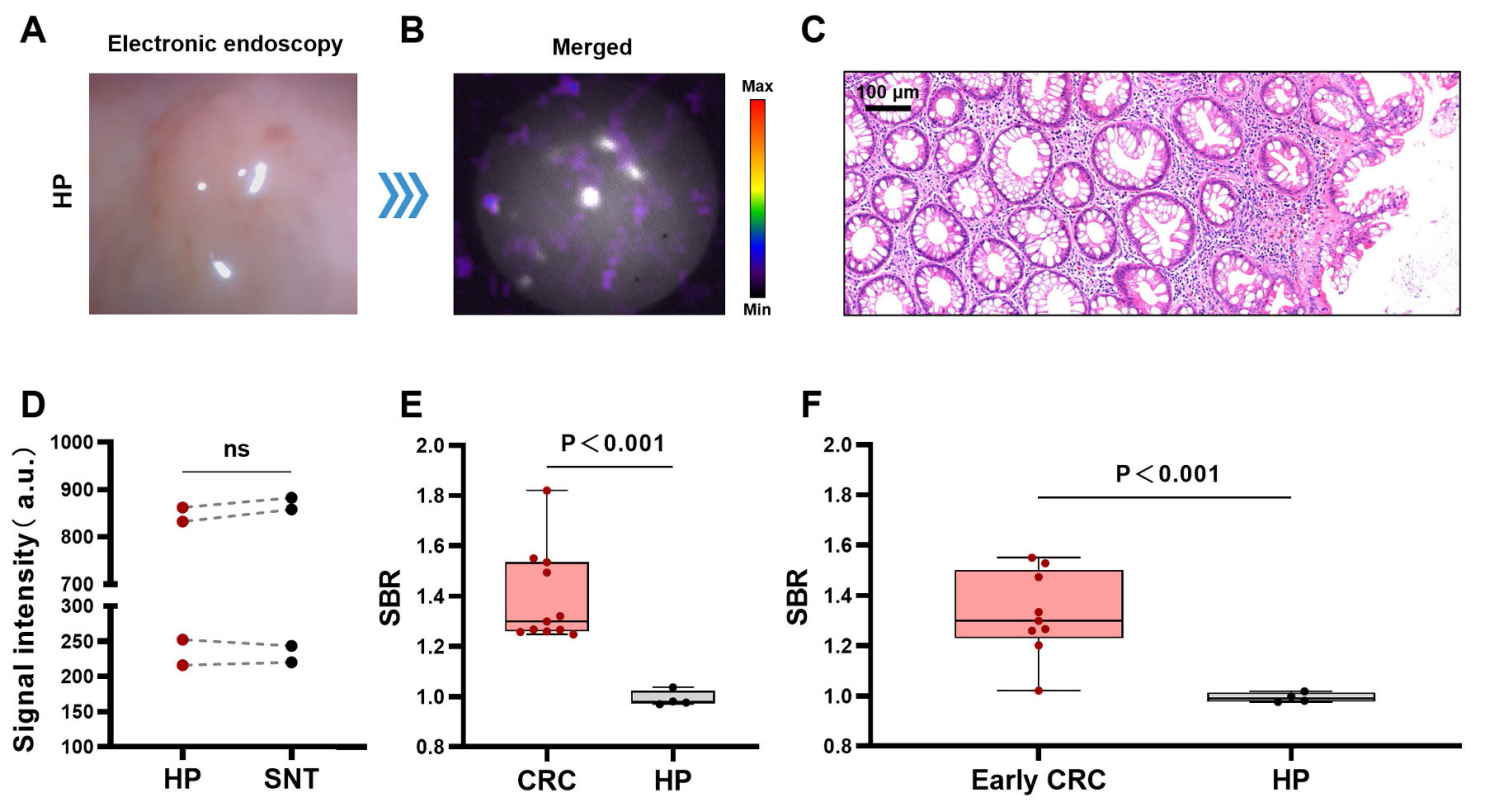
** CLE imaging of HP**. Representative images of CLE ([⁶⁸Ga]Ga-FAPI-04) in the HP (A-B). Pathology image of the HP (C). Comparison of CL signal intensity between HPs and SNTs (t-test, n=4, D). Comparison of the SBRs of the CL signal images of CRC (stage I-IV) and HP (t-test, E). Comparison of the SBRs of the CL signal images of early CRC (stage I CRC and advanced adenoma) and HP (t-test, F). CLE, Cerenkov luminescence endoscopy; CRC, colorectal cancer; SNT, surrounding normal tissue; HP, hyperplastic polyp; ns, not significant.

Hyperplastic polyps are benign non-tumorous lesions and are managed differently from CRCs. However, it may not be easy to distinguish early CRCs from hyperplastic polyps by gross morphology alone under traditional gastrointestinal endoscopy 29, 30. Therefore, we further explored the ability of the CLE system to differentiate CRCs from hyperplastic polyps. Figures 4A-B show a typical CLE imaging result of a hyperplastic polyp approximately 0.3 cm in size of the bowel. The signal intensity of the lesions was similar to that of the surrounding normal tissue. Figure 4C shows the benign histological features of the lesion. The results of the quantitative analysis (n=4) revealed that there was no significant difference in the signal values between the hyperplastic polyps and surrounding normal tissue (*P* = 0.39) (Figure 4D). Compared with hyperplastic polyps, CRCs (stage I-IV) and early CRCs (stage I CRC and advanced adenoma) had greater SBRs (CRCs (stage I-IV), 1.39 ± 0.18; early CRCs, 1.33 ± 0.17; hyperplastic polyps, 0.99 ± 0.03) (Figures 4E-F).

### Agreement between CLE images and histopathology

The consistency of the localization of histologically confirmed malignant lesions (stage I-IV CRC and advanced adenoma) in white light images and CL images was evaluated via a Likert scale. For 87.5% of all malignant lesions, CL images localized the lesion with an "acceptable to high degree of concordance" (≥ 3 on a 1-5 Likert scale) 31. Specifically, 85.7% of the CL images revealed lesions with "acceptable to high agreement" in advanced CRC (stage II-IV), and 88.9% of the CL images revealed lesions with "acceptable to high agreement" in early CRC (stage I CRC and advanced adenoma). Table S3 provides a comprehensive analysis.

### Radiation dose assessment

Radiation dose assessment was performed on the endoscopist and the nurse in contact with the patient. The endoscopist was responsible for operating the CLE system to examine the bowels of the patients and take pictures of the lesions. The nurse was responsible for cooperating with the endoscopist in endoscopic operations. The natural background radiation dose rate in our experimental area is 0.13 μSv/h. The average activity of the initially injected nuclides was 7.09 ± 1.23 mCi. The average operation time for an endoscopist and a nurse to complete one CLE system imaging is 18 ± 8 minutes. The average dose rate for an endoscopist is 20.67 ± 13.64 μSv/h, and for a nurse, it is 7.10 ± 5.06 μSv/h. The average radiation dose to the endoscopist for complete one CLE system imaging was 6.20 ± 3.03 μSv, and the average radiation dose to the nurse was 2.13 ± 1.19 μSv. In accordance with the limits recommended by the International Commission on Radiological Protection 32, which are based on the effective dose of not exceeding 50 mSv annually, endoscopists can perform up to 8,064 cases of CLE imaging annually, and nurses can perform up to 23474 cases of CLE imaging in conjunction with endoscopists. Thus, CLE imaging is relatively safe for medical staff in the clinic.

### CLE system imaging for tumor-bearing mice

**Figure 5:**
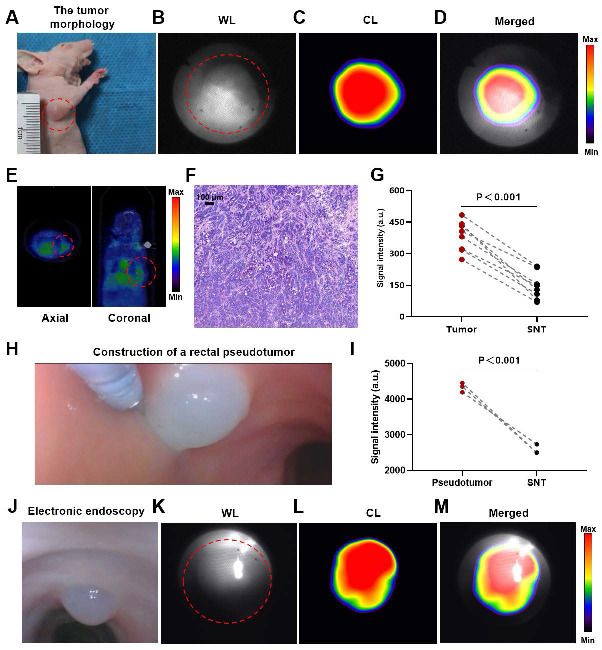
** CLE imaging of mice and dogs**. A representative image of the tumor morphology (red dotted line) (A). The WL photograph of the tumor (red dotted line) (B). CL photograph ([⁶⁸Ga]Ga-FAPI-04) of the tumor (C). Merged image of the tumor (D). PET/CT image ([⁶⁸Ga]Ga-FAPI-04) of the tumor (red dotted line) (E). Pathological image of the tumor (F). ​​Comparative analysis of CL intensity contrasting tumors with SNTs (t-test, n=8, G). Construction of a rectal pseudotumor in the bowel of a dog (H). Comparison of CL signal intensity between the pseudotumor and SNT images (t-test, n=3, I). A representative image of electronic endoscopy for a rectal pseudotumor in the bowel of a dog (J). WL photograph of the pseudotumor (red dotted line) (K). CL photograph of the pseudotumor (L). Merged image of the pseudotumor (M). CLE, Cerenkov luminescence endoscopy; WL, white light; CL, Cerenkov luminescence; SNT, surrounding normal tissue.

The endoscopic lens was aimed at the tumor, and CL imaging was performed. A well-defined CL was detected in the tumor region (Figures 5A-D). PET/CT revealed the same localization result (Figure 5E), confirming the accuracy of CL imaging. The adenocarcinoma histology of the mouse is shown in Figure 5F. The analysis of the quantitative data (n = 8) indicated that the intensity of the signal associated with the tumor was markedly greater compared to the signal intensity observed in the surrounding normal tissues (*P*<0.001) (Figure 5G).

### CLE system imaging for rectal pseudotumor model dogs

After mixing ^68^Ga with Matrigel, we injected the mixture into the rectal mucosal layer of a dog via an endoscopic injection needle to construct a nuclide pseudotumor to mimic a GI tumor under human-like bowel conditions (Figure 5H). Figures 5J-M show a typical case in which a GI pseudotumor was first located via electron endoscopy and then located via fiber-optic endoscopy for CL imaging. The site of the pseudotumor emitted a well-defined CL, which coincided with the actual location of the pseudotumor. The quantitative data (n = 3) revealed that the CL signals of the pseudotumor were significantly higher (*P* < 0.001) compared to the surrounding normal tissue (Figure 5I).

## Discussion

The early diagnosis of CRC is critical for improving the prognosis of patients. However, traditional gastrointestinal endoscope has a relatively low diagnostic rate for early CRC, with less than 20% of CRCs detected at an early stage 33. In addition, most CRCs evolve from adenomas 27, ​​which​​ are often missed during routine colonoscopy—especially flat or small ​​ones​​, with missed diagnostic rates as high as 34% and 28%, respectively 34. CLE, as a molecular imaging technique, can identify small or invisible morphological changes in the mucosa. When we used CLE for imaging CRCs of stage I and advanced adenomas, the CLE system effectively identified these lesions, demonstrating a strong alignment with histopathology and PET/CT, highlighting its potential benefits in the early detection of CRC, especially for early lesions with subtle morphological changes that may be missed by white light endoscopy. Since CLE imaging is a type of molecular functional imaging, it can also distinguish early CRCs from hyperplastic polyps that do not yet have the characteristics of malignant tumors.

The successful implementation of Cerenkov radiation for tumor diagnosis has been reported for some tumors on the body surface 31, 35, 36. In this study, we used a self-developed CLE to perform CL imaging for mucosal tumors and achieved molecular imaging in CRC patients. This is the first study to achieve targeted CL imaging of early CRC in humans, which implies the significant value of CLE for the early diagnosis of gastrointestinal cancers. Moreover, considering that the radiotracer also has good targeting for some superficial solid tumors (such as thyroid cancer and breast cancer) and for other tumors of natural orifices, like cervical cancer and bladder cancer 31, 37, 38, CLE has the potential to be further applied to the early diagnosis of these tumors. Additionally, several studies have reported the application of CLI in intraoperative margin assessment. The use of CLI for margin assessment of *ex vivo* tumors in breast and prostate cancer resections has shown good consistency with histopathology 36, 39. CLE also has the potential to outline the edge of the gastrointestinal tumor, thereby improving the precision of gastrointestinal tumor surgery.

The fundamental drawback of the weak CL emission necessitates a prolonged image acquisition time to achieve reliable detection. Nevertheless, it is crucial to maintain the imaging time within a suitable range, as a long duration of imaging lowers the efficiency and impedes clinical application. In our study, when the image acquisition time was decreased to 60 s, the CLE acquired a similar result without dropping down of the imaging quality from the 300 s CLE. Reducing the imaging time to 60 s represents a significant reduction compared to the imaging times in previous studies (which were at least 5 minutes or more). 31, 40, significantly improving the feasibility and convenience of clinical applications and making it possible to detect tumors in other parts of the digestive tract.

CLE imaging is a functional molecular imaging technique. The intensity of the CL signal emitted by benign hyperplastic colorectal polyps is not different from that of the surrounding normal tissue, i.e., negative imaging under the CLE system. With this feature of CLE, CRC or advanced adenoma (some are difficult to distinguish from hyperplastic polyps during conventional gastrointestinal endoscopy 29, 30) can be effectively differentiated from hyperplastic polyps that do not yet have the molecular characteristics of malignant tumors. In an ideal situation in the future, for a small lesion, if the CLE diagnosis considers it to be a hyperplastic polyp, additional examinations and treatments may not be necessary. However, if it is considered a malignant lesion, surgical resection will be required. Moreover, even for large lesions, the subsequent examinations and treatment methods for benign and malignant lesions are different. Benign lesions only require local resection and do not need additional examinations, while malignant lesions necessitate further tests to clarify the depth of lesion invasion, whether there is metastasis, etc. Of course, the ability of CLE to differentiate between benign and malignant lesions will need to be validated through larger sample sizes in the future.

To improve the quality of CL imaging, the higher intensity of CL emitted by ^68^Ga rather than ^18^F is often selected on the basis of previous in vitro studies 24, 41. We compared two nuclide probes, [¹⁸F]FDG and [⁶⁸Ga]Ga-FAPI-04, as contrast agents. [¹⁸F]FDG is a classical nuclide probe. As a glucose analog, [¹⁸F]FDG can be transported into cells via glucose transporters on the cell membrane. Tumor cells have a vigorous metabolism and often express high levels of glucose transporters, so they can take up more [¹⁸F]FDG than normal cells 15, 42. [⁶⁸Ga]Ga-FAPI-04 is a new type of nuclide probe that was recently developed to target epithelial tumors. It targets fibroblast activation protein (FAP), which is highly expressed in cancer-associated fibroblasts (CAFs) in the tumor stroma, to achieve high-concentration accumulation in tumor tissue 43. In our clinical study, there was no significant difference in the diagnostic rate or imaging quality of CRC between [¹⁸F]FDG and [⁶⁸Ga]Ga-FAPI-04, which could be extended to the feasibility of the clinical application of CLE.

To evaluate the exposure risk of this procedure, we also measured the radiation dose to the relevant operators during a single imaging session of the CLE system. Even the endoscopist who received the highest radiation dose only registered approximately 6 μSv, which is less than one-thousandth of an ordinary chest CT 44. According to international standards, an endoscopist can safely complete at least 8,000 CLE imaging operations per year at this dosage rate.

We also performed several preclinical experiments before the clinical trial. The phantom experiment confirmed that the light emitted by the nuclide probe is indeed CL. Imaging experiments in nude mice revealed that the CLE system could detect the CL in the tumor region. Rectal pseudotumor imaging experiments in dogs also revealed that the CLE system could detect CL emitted by a nuclide pseudotumor and precisely locate it in the entire light-free digestive tract in vivo. The above results showed that the CLE system could detect CL in tumors or tissues. Additionally, the gastrointestinal tract is indeed a naturally good light-shielding environment for CL imaging. These preclinical results lay the foundation for developing clinical trials for CLE.

There are several limitations of this study. Owing to the short wavelength of the CL, absorption and scattering phenomena occur when the CL penetrates tissues 45, 46, reducing the intensity of the CL and leading to some inaccuracy in CLE localization. Research on shortwave infrared CL has demonstrated its superior tissue penetration capabilities, which implies that it functions to locate objects precisely in deep tissues 47. Further research should further explore the potential of shortwave infrared CL. In addition, although we have shortened the imaging time of the CLE to 60 s, which is an improvement over previous studies 31, 40, the CLE is not yet capable of real-time imaging, which can prolong the endoscopic examination time for patients and lead to operator inconvenience. We are in the process of constructing a more sensitive CLE using a new nanomaterial that can significantly increase the intensity of radionuclide luminescence, dramatically reducing imaging time. Real-time imaging is promising in the future.

In conclusion, our study reports for the first time that a self-produced CLE can utilize molecular imaging to diagnose and differentiate early CRC, overcoming the limitations of current endoscopic diagnosis based on morphological changes. The CLE holds promise to enhance the detection rate of early CRC by endoscopists.

## Supplementary Material

Supplementary methods and tables.

## Abbreviations

CAF: cancer-associated fibroblast
CL: Cerenkov luminescence
CLE: Cerenkov luminescence endoscopy
CRC: colorectal cancer
EMCCD: electron-multiplying charge-coupled device
FAP: fibroblast activation protein
SBR: signal-to-background ratio
