# Supplementary material for: Early diagnosis of colorectal cancer using Cerenkov luminescence endoscopy: a pilot trial involving humans for the first time
Source: Theranostics. 2026 Jan 8;16(7):3685–96. doi: 10.7150/thno.122007 (PMC12846752; doi:10.7150/thno.122007)
Supplement: Supplementary file 1 — Supplementary methods and tables. [file thnov16p3685s1.pdf]

Supplementary Materials

CONTENTS

1. SUPPLEMENTARY METHODS 1
Phantom imaging study 1
CLE imaging of tumor-bearing nude mice 1
CLE imaging of rectal pseudotumor model dogs 1
Hematoxylin-eosin staining 2
CL image postprocessing process 2
2. SUPPLEMENTARY TABLES 3
Table S1. The main parameters of the Cerenkov luminescence endoscopy system 3
Table S2. Characterization of 20 colorectal mucosal lesions in 15 patients 4
Table S3. Agreement between CRC images of CLE and histopathology 5

1. SUPPLEMENTARY METHODS

Phantom imaging study

A standardized resolution test board (USAF 1951, USA) was used to characterize the white light resolution. The test board has six sets, and each set of line pairs contains six elements. Each set of line pairs with different elements corresponds to a different resolution. The white light resolution was calculated as described previously [1].

Cerenkov luminescence (CL) confirmation experiments: Approximately seven μCi of ^{68}Ga solution was added to one well of a black 96-well plate as a light source. The endoscopic lens was first aimed at the nuclide well to take a picture, and then a piece of black cardboard covered the nuclide well. All other conditions remained unchanged, the imaging continued, and the procedure was repeated three times.

Comparison of Cerenkov luminescence endoscopy (CLE) with commercial optical imaging system: The commercial optical imaging system utilized the small animal in vivo imaging system (IVIS Lumina S5, PerkinElmer, USA). A mixture of ^{68}Ga and physiological saline with a total volume of 300 μl was injected into the round wells of a black 96-well plate as the CL source. Both CLE and IVIS Lumina S5 were used to image the CL source with the same radionuclide activity, with an imaging time set at 300 s and a binning value of 4. The experiment was repeated 7 times.

CLE imaging of tumor-bearing nude mice

The mice used in this experiment were of the BALB/c-nu strain (Charles River Laboratories, Beijing). They were housed in a specific pathogen-free (SPF) environment with an indoor temperature of approximately 22 ℃, relative humidity of about 60%, and a 12-hour light/dark cycle. The mice had free access to food and water. Following the cultivation of HT29 cells (Zhongqiao Xinzhou Biotechnology Co., Ltd., China) until they reached the logarithmic growth phase, the tumor cells were injected into the right upper limb near the trunk side of the female nude mice (4–6 weeks old), the total volume of injected cells per nude mouse was 0.1 ml, the number of cells was 2×10^6 cells, and imaging experiments were performed when the diameter of the tumors reached 6–8 mm. For the approximate imaging procedure, tumor-bearing nude mice were injected with approximately 50 μCi of ^{68}Ga-FAPI-04 via the tail vein. After approximately 30 min, CLE imaging was performed in a light-avoidance environment.

CLE imaging of rectal pseudotumor model dogs

The dogs used in this experiment were Chinese rural dogs raised in the animal room of the Department of Experimental Surgery at the First Affiliated Hospital of Air Force Medical University.

The indoor temperature was approximately 24 °C, relative humidity was about 50%, and there was a 12-hour light/dark cycle. The dogs had free access to food and water. The rectal pseudotumor model of dogs was constructed as follows: ^{68}Ga was mixed with Matrigel (HY-K6007, MCE, Monmouth Junction, NJ, USA), and the mixture was injected into the rectal mucosal layer of the dog through an endoscopic injection needle to construct a nuclide pseudotumor. A CLE was inserted via the anus of the dog, and the electronic endoscope was prepositioned, followed by CL imaging.

Hematoxylin-eosin staining

Hematoxylin-eosin staining was performed on 4-μm-thick, routinely processed paraffin-embedded sections. The paraffin sections were dewaxed by placing them in xylene I/II for 8 min each, followed by 100% ethanol I/II, 95% ethanol, 85% ethanol, and 75% ethanol for 5 min each, and then washed with distilled water. The sections were stained with hematoxylin for approximately 5 minutes and then rinsed under running water. They were differentiated in hydrochloric acid and alcohol for a few seconds and washed under running water for 5 min. The sections were then stained with eosin for 1 min. The sections were placed in 75% ethanol for 30 s, 80% ethanol for 30 s, 95% ethanol I/II for 1 minute each, 100% ethanol I/II for 5 minutes each, and xylene I/II for 5 minutes each. The sections were then air-dried and mounted with neutral gum. Finally, the sections were observed and photographed under a microscope (Olympus, Japan).

CL image postprocessing process

The raw images were processed via ImageJ (1.52a). The initial measurement of the signal value within the area of interest was conducted on the original image. Anomalous signals caused by gamma rays were subsequently removed via the remove outliers function. The bandpass FFT function further removed the high spatial frequencies and low spatial frequencies, and the obtained image was then median filtered. The LUT function was utilized to pseudocolor the image. The resulting image was manually adjusted if necessary.

2. SUPPLEMENTARY TABLES

Table S1. The main parameters of the Cerenkov luminescence endoscopy system

Type of equipment	Instrument features	Performance parameters
Electronic endoscope	Resolution	0.035 mm
	Pixel count	2 million
	Video format	1080 P
	Frame Rate	30 fps
Fiber-optic endoscope	Resolution	0.315 mm
	In vitro sensitivity	0.012 µCi (radionuclide: ^{68}Ga, imaging time: 300 s)
	In vivo sensitivity	0.020 µCi (radionuclide: ^{68}Ga, imaging time: 300 s)
EMCCD	Sensor format	512 x 512
	Pixel Size	16 µm
	Frame Rate	56 fps
	Read Noise	< 1 e- with EM Gain
	Pixel well depth	180,000 e-

EMCCD, electron-multiplying charge-coupled device.

Table S2. Characterization of 20 colorectal mucosal lesions in 15 patients

Patient No.	Histopathology	TNM Stage (Prognostic Groups)
1	Moderately differentiated adenocarcinoma	pT2N0M0(I)
2	Moderately differentiated adenocarcinoma、mucous adenocarcinoma	pT3N0M0(IIA)
3	Moderately differentiated adenocarcinoma	cT2N0M0(I)
4	Well differentiated adenocarcinoma	ypT3N0M0(IIA)
	Hyperplastic polyp	NA
5	Moderately differentiated adenocarcinoma	cT4aN2M1a(IVA)
6	Moderately differentiated adenocarcinoma	cT4aN0M1b （IVB）
7	Hyperplastic polyp	NA
	Moderately differentiated adenocarcinoma	pT2N1aM0(IIIA)
8	Tubulovillous adenoma	NA
9	Tubulovillous adenoma	NA
10	Moderately differentiated adenocarcinoma	pT1N0M0(I)
11	Moderately differentiated adenocarcinoma	cT3bN0M0 (IIA)
	Tubulovillous adenoma	NA
	Hyperplastic polyp	NA
12	Moderately differentiated adenocarcinoma	pT3N0M0(IIA)
13	Tubulovillous adenoma	NA
14	Moderately differentiated adenocarcinoma	pT1N0M0(I)
	Hyperplastic polyp	NA
15	Traditional serrated adenoma with high-grade intraepithelial neoplasia	NA

NA, not available.

Table S3. Agreement between CRC images of CLE and histopathology

Score	Advanced CRC	Early CRC	Total
No (%)	7	9	16
5 scores	3(42.9)	3(33.3)	6(37.5)
4 scores	2(28.6)	4(44.4)	6(37.5)
3 scores	1(14.3)	1(11.1)	2(12.5)
2 scores	1(14.3)	0(0.0)	1(6.3)
1 score	0(0.0)	1(11.1)	1(6.3)
4-5 scores	5(71.4)	7(77.7)	12(75)
3-5 scores	6(85.7)	8(88.9)	14(87.5)
1-2 scores	1(14.3)	1(11.1)	2(12.5)

Individual counts and percentages reflect Likert scores. Likert scores: 1, strongly disagree with correlation; 2, disagree with correlation; 3, intermediate or neutral correlation; 4, agree with correlation; 5, highly agree with correlation. CRC, colorectal cancer; CLE, Cerenkov luminescence endoscopy.

REFERENCES

1. Cao X, Chen X, Kang F, Lin Y, Liu M, Hu H, et al. Performance evaluation of endoscopic Cerenkov luminescence imaging system: in vitro and pseudotumor studies. Biomed Opt Express. 2014; 5: 3660.
